# Effects of Scooping Mobilization and Muscle Energy Technique on Pain and Range of Motion in Post-fracture Elbow Stiffness: A Case Report

**DOI:** 10.7759/cureus.31141

**Published:** 2022-11-05

**Authors:** Priya R Chandak, Pooja Dhage, Nikita S Deshmukh

**Affiliations:** 1 Department of Musculoskeletal Physiotherapy, Ravi Nair Physiotherapy College ,Datta Meghe Institute of Medical Sciences, Wardha, IND; 2 Department of Musculoskeletal Physiotherapy, Ravi Nair Physiotherapy College, Datta Meghe Institute of Medical Sciences, Wardha, IND

**Keywords:** muscle energy technique ( met ), muscle energy technique, scooping mobilization, acute pain, physiotherapeutic rehabilitation, extensor lag of elbow, upper extremity fracture, elbow stiffness

## Abstract

Olecranon fractures are breaks in the elbow's bony tip. One of the three bones that come together to form the elbow joint is the ulna, which is made up of this pointed section of bone. The olecranon prevents the ulna from anteriorly translating relative to the terminal humerus. Olecranon fractures can result either indirectly or directly from trauma. The posterior point of the elbow may be directly fractured following a fall or severe injury. Extreme activation of the triceps muscle following a fall on a partly extended elbow may result in indirect avulsion of the olecranon. First, the most typical method of identifying a fracture is with the use of an x-ray, while magnetic resonance imaging (MRI) allows for the detection and confirmation of soft tissue involvement. Physiotherapeutic treatment assists in pain relief, extending the range of motion, and quick healing. Early intervention after open reduction and internal fixation reduces post-fracture stiffening. The readership of the research study is intended to be informed regarding the various treatments available for elbow extensor lag and post-fracture elbow stiffness. This report offers suggestions to improve the patient's care and functionality after an olecranon fracture.

## Introduction

Olecranon fractures, olecranon fracture-dislocations, and coronoid process fractures are examples of proximal ulna fractures [1]. Frequent upper-limb accidents, such as olecranon fractures, are frequently brought on by a forceful hit to the elbow following a drop from a standing position. Less frequently, individuals suffer incidental strain damage from the triceps connection. The olecranon and coronoid processes compose the larger sigmoid gap and are divided by a region of the joint region devoid of hyaline cartilage, sometimes known as the "bare spot" [2]. Olecranon fractures constitute 10% of all upper extremity lesions. The lesion may be the result of indirect or direct trauma, especially forced hyperextension of the elbow joint. Several classification systems have been developed, but none is generally accepted [2]. Post-traumatic elbow stiffness has been classified according to the components involved or the anatomic location. Olecranon fractures are classified according to the Mayo classification into types I-III depending on the degree of stabilization and dislocation visible in imaging studies; 5% of all cracks are Type I, which are non-displaced. Eighty to eighty-five percent of olecranon fractures are Mayo type II fractures, which are displaced and show an intact medial collateral ligament, especially the anterior portion. Type III injuries feature a dislocated olecranon fracture with the instability of the humeral joint and damaged collateral ligaments. Each type is further subdivided into two types: (a) non-comminuted and (b) comminuted. Around 5% of all fractures are type I, which are non-displaced. Type II fractures of olecranon cracks are shifted with a solid no humeral joint, denoting preserved soft-tissue formations, especially the anterior fraction of the medial collateral ligament. In type III damage, the ulno-humeral joint is displaced, suggesting that the collateral ligaments were ruptured [3]. In the past, most instability incidents were treated invasively to reunite the triceps tendon and restore the uniformity of the ulnar anatomic surfaces [4].

## Case presentation

Patient information

A 52-year-old female patient, a homemaker, came to the OPD with the chief complaints of pain over the right elbow, swelling, and restricted range of motion. She had a history of falling in the bathroom over her right elbow. She immediately visited the nearby hospital, where investigations were done and she was diagnosed with an olecranon fracture. For the same conservative management, an above-elbow plaster of Paris (POP) cast for one month was advised. After one month of conservative management, she developed elbow stiffness. After two weeks, she again had an accidental fall and was injured over the same elbow, for which medication was given. Further, due to her negligence regarding the injury, she developed elbow stiffness and extensor lag in the elbow.

Clinical findings

On examination, the patient had a mesomorphic build with a medical history of hypertension for five years and diabetes mellitus with ongoing medication. All the vital parameters were normal. On local examination, there was severe pain over the olecranon process and a decrease in the elbow's range of motion. A numerical pain rating scale score of 8/10 for movement and 5/10 for rest is shown in Table 1.

**Table 1:** Numerical pain rating scale (NPRS) score indicates before and after the assessment of pain.

NPRS	On assessment	After two weeks
On activity	8/10	3/10
On rest	5/10	0/10

The swelling was present over the elbow. On palpation, there was soreness over the olecranon process, indicating grade 2 tenderness; a reduced and painful active and passive range of motion for elbow flexion, extension, pronation, and supination are shown in Table 2. The range of motion of the wrist was also affected, as shown in Table 2. Manual muscle testing for all the elbow muscles tested received a 3/5 rating, before and after the assessment, as shown in Table 3. The numerical pain rating scale (NPRS) score indicates the level of pain before and after the assessment. The numerical pain rating scale (NPRS) score indicates the level of pain before and after the assessment.

**Table 2:** The range of motion (ROM) of the right upper limbs before and after the assessment in degrees. ROM- range of motion.

Range of motion	On assessment - active ROM	On assessment - passive ROM	After 2 weeks - active ROM	After 2 weeks - passive ROM
Elbow flexion	0-39	0-55	0-145	0-150
Elbow extension	39-16	55-03	145-0	150-0
Supination	0-28	0-32	0-69	0-74
Pronation	0-31	0-43	0-81	0-85
Wrist flexion	0-68	0-69	0-76	0-80

**Table 3:** Manual muscle testing (MMT) indications before and after the assessment.

MMT	On assessment	After two weeks
Elbow flexion	3/5	5/5
Elbow extension	3/5	5/5
Pronation	3/5	5/5
Supination	3/5	5/5
Wrist flexion	4/5	5/5
Wrist extension	4/5	5/5

Therapeutic intervention

Scooping Mobilization

**Figure 1:**
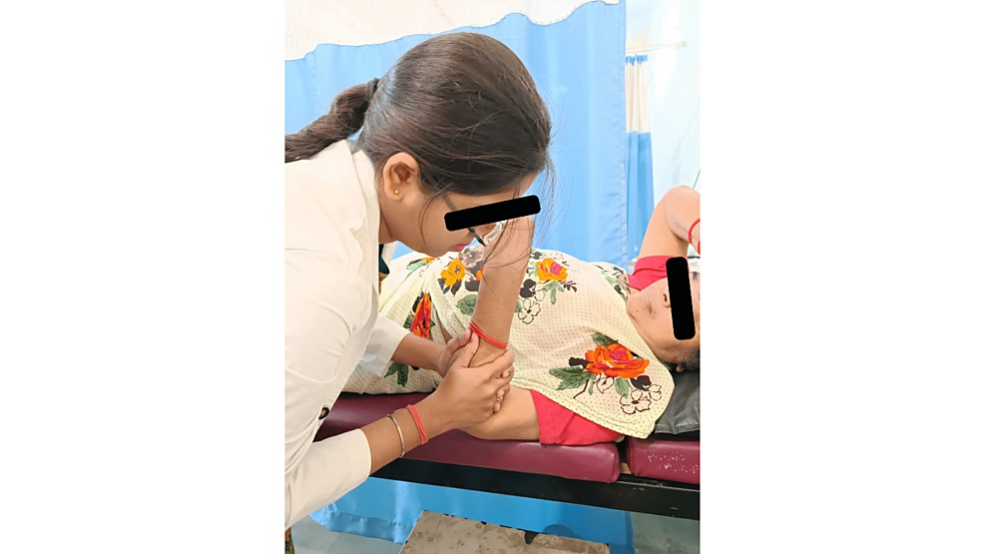
Scooping technique for the elbow.

The procedure used to lessen discomfort and expand the joint's range of motion is called joint mobilization. The bulk of joint troubles is treated with the procedure [5]. The patient was positioned supine, lying with the elbow at the side of the table. In the primary procedure, the joint was held in a relaxed posture, and the client's hand rested on the therapist's shoulder. The physiotherapist placed the elbow at the limit of the range before stretching into flexion or extension. The patient's elbow was maintained at the maximum amount of flexion possible. The physiotherapist positioned one hand on the volar exterior over the proximal part of the ulna and then strengthened it using the other hand. Force-directed as a distraction, it was initially applied by the physical therapist at an angle of 45° to the ulna, followed by a distal orientation along the longitudinal plane of the ulna while retaining the distraction force. It is referred to as the "scooping technique," which is shown in Figure 1.

Muscle Energy Technique (MET)

**Figure 2:**
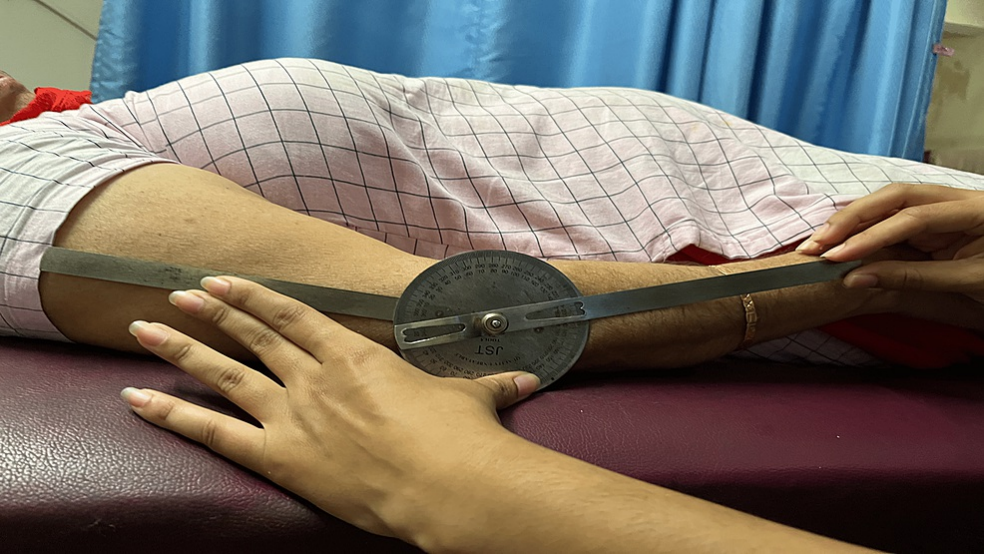
The range of motion before assessment.

**Figure 3:**
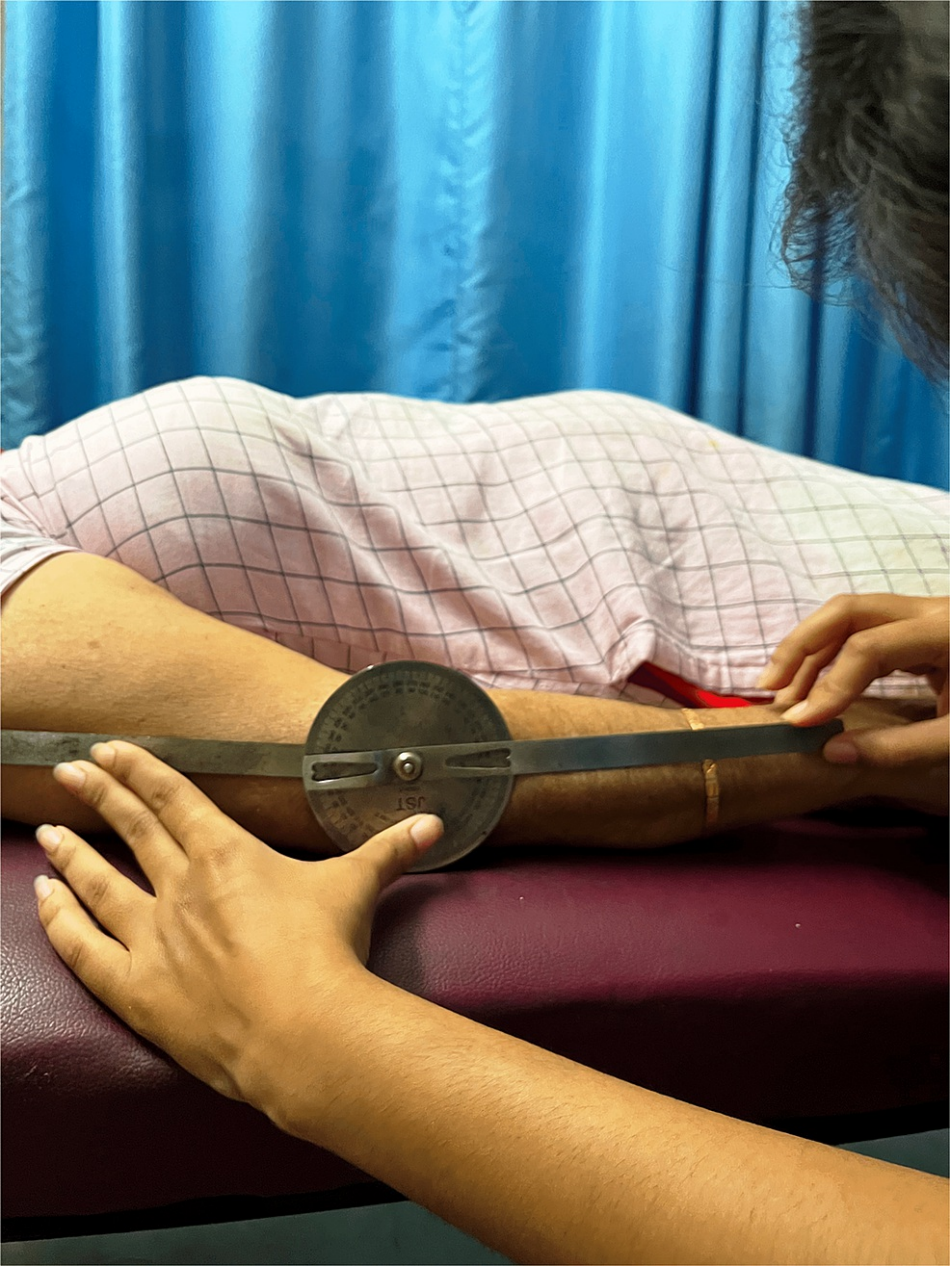
The Range of motion after assessment.

MET is a series of mobilization procedures that are performed to recover mobility, decrease tissue swelling, ease muscular spasms, lengthen the tissue, and teach the intersegmental related muscles' stabilizing abilities. In MET, the patient contracts the muscle voluntarily and with exact control in opposition to the therapist's counterforce. These treatment results include pain relief, improved muscle tone, muscular stretching, muscle strengthening, increased local blood flow, and joint limitation mobilization. In this procedure, the patient's muscle was actively contracted, and the muscle was relaxed. The hypertonic muscle was stretched to the point where movement opposition will be initially felt or just past the level of discomfort. About five to ten seconds were spent doing a submaximal (10%-20%) contraction of the hypertonic muscle far from the barrier while the physiotherapist provided resistance in a reverse manner. Throughout this attempt, the client was told to breathe in. The patient was instructed to unwind and breathe out as they did following the isometric contraction. An easy stretch could then be used to take up the slack until the new barrier was in place. The process was then carried out again (three times), beginning at each new limit. The dosage was given for three sets and 15 repetitions. Figure 2 shows the range of motion of the patient before the treatment, and Figure 3 shows an increase in the range of extension after the treatment.

Ultrasound

Ultrasound is a popular non-invasive treatment technique in the physiotherapy industry. Connective tissue is principally responsible for absorbing the waves. Therapeutic ultrasound's thermal and non-thermal effects cause biochemical reactions such as muscle calming, tissue repair, and a reduction in inflammation [6]. Ultrasound therapy was given at a dosage of 3 MHz, 1.5 W/cm2, and in pulsed mode for 1 ms on and 5 ms off. The area of the transducer head was 4 cm2. The treatment head was kept in motion, and skin contact was at the palpated point [7].

Strengthening 

Isometric contractions: The elbow of the damaged arm was bent to 90 degrees, and the left hand of the uninjured arm applied physical traction over the dorsal side of the supinated hand of the unaffected arm. The wrist extensors were isometrically contracted and maintained for about five to ten seconds. Active concentric and eccentric exercises: When the distress diminishes, most of the exercises are introduced. With the elbow tilted to 90 degrees and the hand hanging over the rim of the bed, the forearm was resting on a table. The wrist was both flexed and relaxed. After keeping the wrist focused and extended for five seconds, eccentric extensor work gradually reduced the hand back to its relaxed starting spot. Dosage: 10 repetitions, twice per day [7]. Progression: The patient was told to perform all repetitions and then gradually increase the weight. The training evolved under distress-free boundaries.

## Discussion

Removing scar structures and fostering a return to baseline functioning after soft tissue repair are indeed the main objectives of mobilization. It lessens the suppleness of the tissue and induces friction, which can create stress and reduce soft tissue activity. Scar tissue, in particular, hinders collagen production and tissue repair, inhibits perfusion to the wounded soft tissue, and restricts the availability of oxygen and minerals. This might result in inadequate treatment outcomes [8]. Using it shortly after three weeks of immobility and one week after the table, the muscle energy technique was achieved. The Golgi tendon organ is triggered by a powerful muscular contraction during progressive isometric relaxation in the face of a similar counterbalance. A blocking motor neuron is encountered by the afferent neurons' signal from the Golgi tendon organ as it reaches the dorsal root of the spinal column [9]. As a consequence, the muscle tone lowers, which causes the agonist to relax and elongate. This also inhibits the release of the efferent motor neurons' impulse, preventing continued contraction. It seems that acute soft tissue injuries, the reduction of inflammation, and the discomfort related to these illnesses are the principal uses for which physiotherapists employ ultrasound. Frequently, it is applied to the management of slow-healing wounds. help speed up bone healing, remove scar tissue and fascia, and accelerate the repair of bone [10]. Mostly in early research, it was shown that ultrasound may lessen pain and swelling in combination with phonophoresis. In the second double-blind study, it was shown that mock ultrasound, or ultrasound that wasn't used through treatment, similarly reduced discomfort, edema, and the initial stage reactant C-reactive protein [11].

## Conclusions

Physiotherapy is a crucial component of treating elbow extensor lag during rehabilitation. Numerous studies have proven successful in correcting elbow extensor lag, yet nobody has demonstrated a significantly increased range of extension in a shorter period. Providing a scooping technique will increase elbow flexor range. To increase elbow extension, a muscle energy technique is administered, and ultrasound is used to relieve pain and discomfort. These are the interventions delivered to patients that achieve desirable, more positive effects immediately. This procedure can also be implemented in future therapeutic management because it is beneficial for resolving extensor lag of the elbow and post-fracture elbow stiffness.
